# Supplementary material for: Molecular detection and antimicrobial resistance profiles of Extended-Spectrum Beta-Lactamase (ESBL) producing Escherichia coli in broiler chicken farms in Malaysia
Source: PLoS One. 2023 May 19;18(5):e0285743. doi: 10.1371/journal.pone.0285743 (PMC10198488; doi:10.1371/journal.pone.0285743)
Supplement: S1 File — (DOCX) [file pone.0285743.s002.docx]

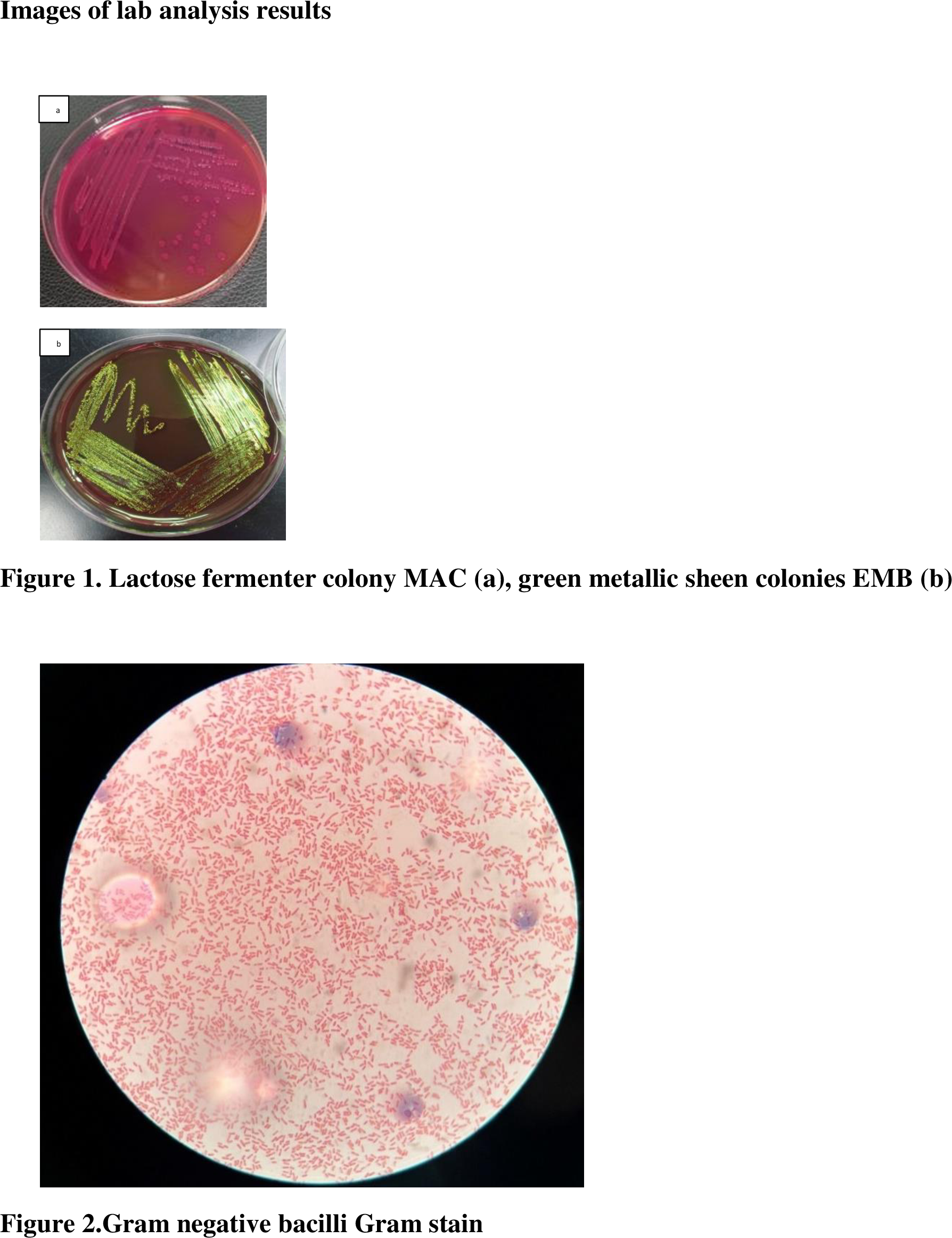


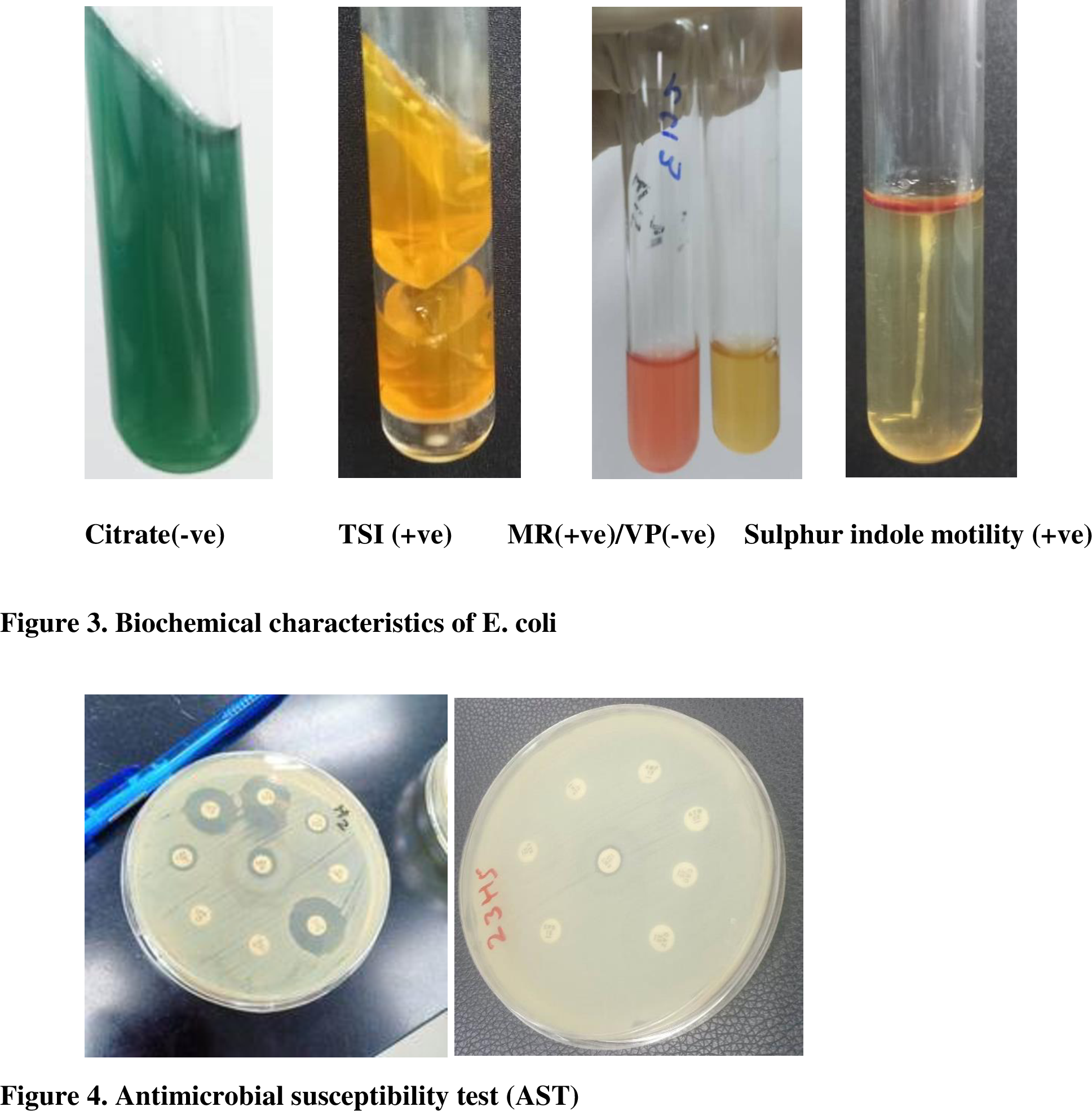


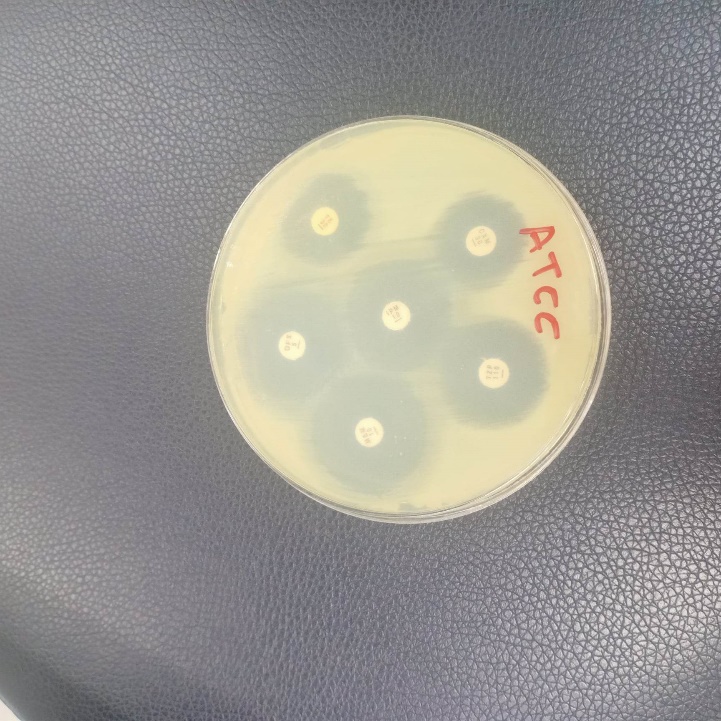


**Figure 5.** *E. coli* ATCC^®^ 25922 Quality control of AST


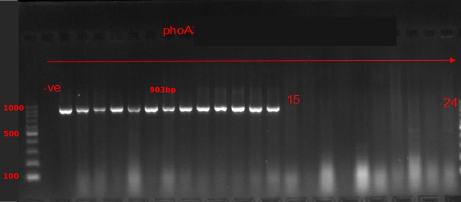


**Figure 6. *Pho* gene(903bp)**


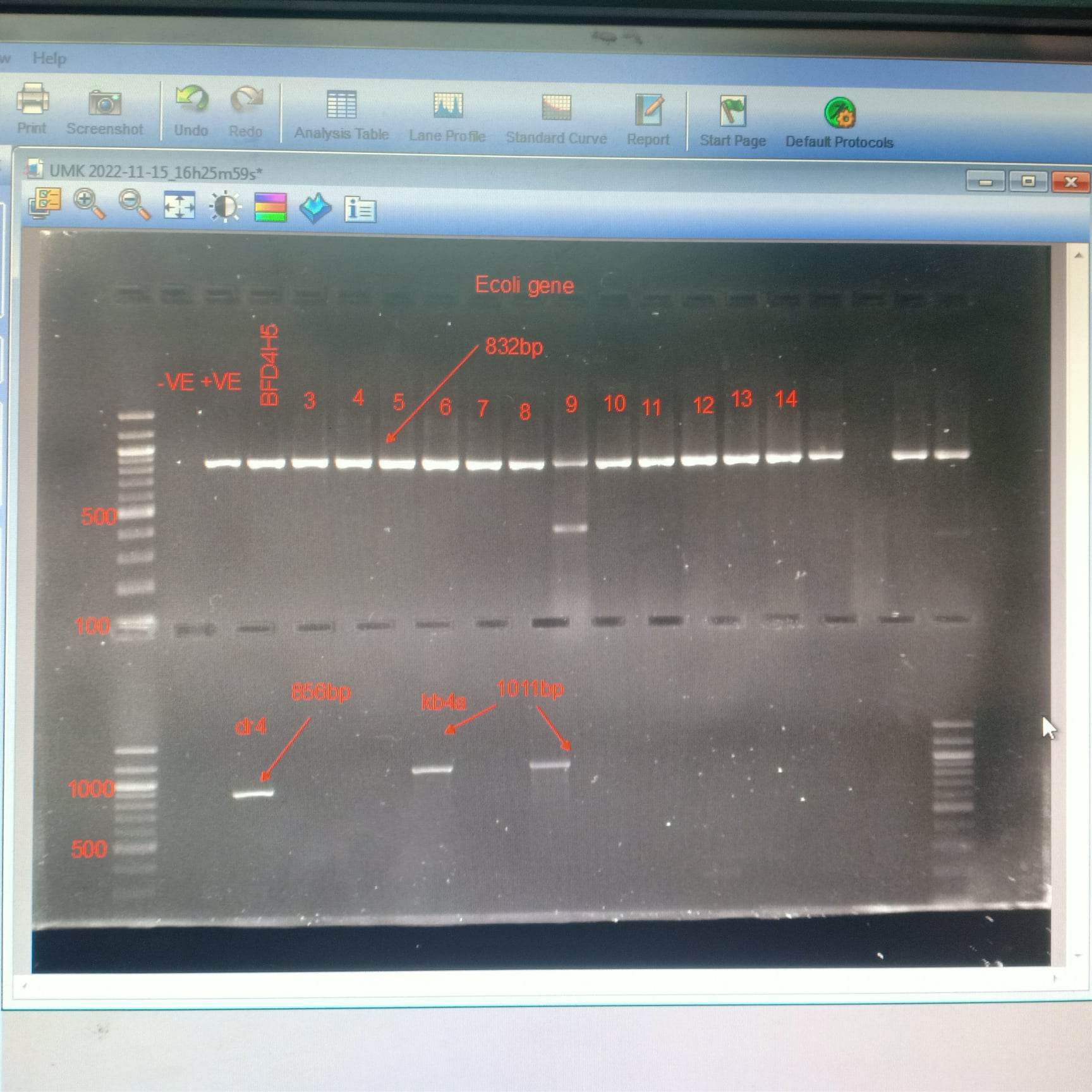


**Figure 7*. E.coli* gene (832bp)**


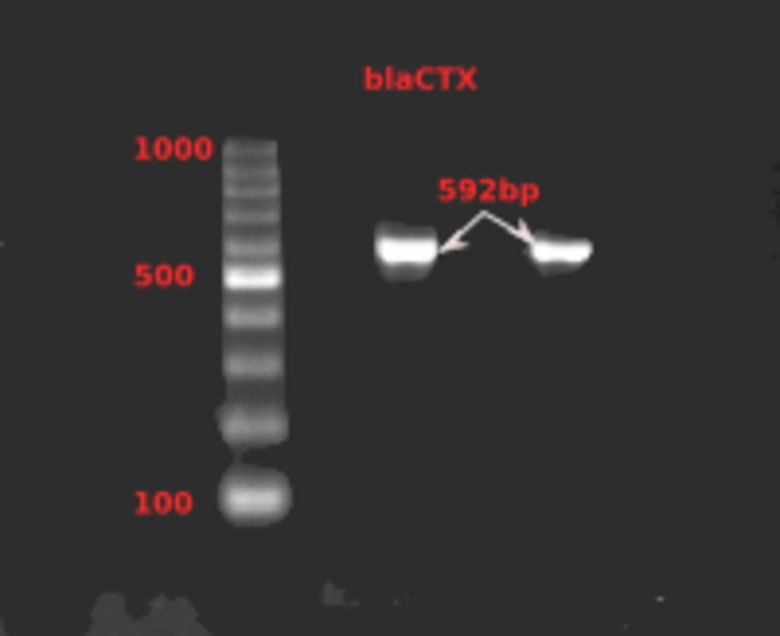


**Figure 8. *bla_CTX-M_* (592bp)**

***
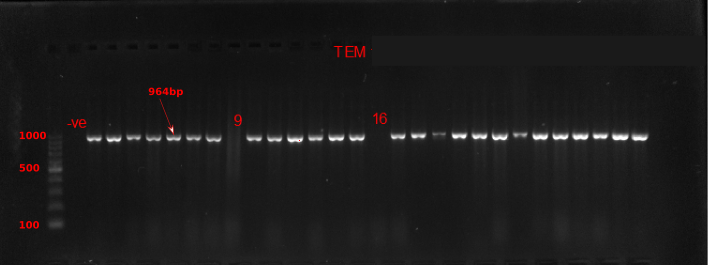
***

**Figure 9*. bla_TEM_* (964bp)**

***
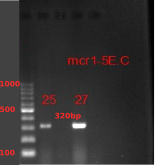
***

**Figure 10. *mcr-1* (320bp)**
