# Supplementary material for: Molecular detection and antimicrobial resistance profiles of Extended-Spectrum Beta-Lactamase (ESBL) producing Escherichia coli in broiler chicken farms in Malaysia
Source: PLoS One. 2023 May 19;18(5):e0285743. doi: 10.1371/journal.pone.0285743 (PMC10198488; doi:10.1371/journal.pone.0285743)
Supplement: Attachment — Submitted filename: PONE-D-22-25669-Review comments-Salequl.docx [file pone.0285743.s004.docx]

# “Molecular Detection and Antimicrobial Resistance Profiles of Extended-Spectrum Beta-Lactamase (ESBL) Producing Escherichia coli in Broiler Chicken Farms in Malaysia”

# Review comments by Md. Salequl Islam

The above entitled manuscript by Mulu Lemlem Desta et al., evaluated the antimicrobial resistant profiles of *Escherichia coli* bacteria isolated from broiler chicken farms in Malaysia.

The manuscript is well-written. I suggest the following points to be considered before deciding this manuscript for publication:

1. Page 11, Line 102-03: Please provide brief description how Multiple antibiotic resistance (MAR) index was analyzed.

2. Some anomalies of reference citation were noticed. Reference number # 27 and #28 are the publication shown differently. Similar more anomalies might be present. Please provide some other stronger references that attest 'Pho-gene" specific *E.coli* detection. Please include 16s rDNA gene sequence to confirm *E. coli*.

3. Please provide a supplementary figure stating cultural, biochemical characteristics, antimicrobial susceptibility analyses, and Polymerase chain reaction results for different genes described in the manuscript.

4. At page 14, line 140-41, described higher resistance of the *E. coli* isolates against meropenem (28.9%) than that of imipenem (17.5%) is unusual. Need to reinvestigate the findings.

5. Table 2 has described both the resistant and susceptible frequency. As resistant frequency will be the complimentary of the susceptible frequency considering the total number tested, describing both frequencies are unnecessary.

6. Page 15-16, line 150-162, as part of ESBL gene analysis only two genes, bla-TEM and bla-CTXM were analyzed.

a) Some more genes should be included to draw conclusion about ESBL associated resistance.

b) There was no description about the type of bla-CTXM (type-9/ 15?).

c) There should have statistical analyses see the association of the bla-TEM and bla-CTXM genes with the bacterial phenotypic resistance phenomena.

d) Not clear why colistin resistance gene, mcr-1 was analyzed under the titleship of ESBL. Need justification at the introduction and discussion.

7. For performing AST, authors should use at least one or two ATCC control to validate and interpret their result.

8. This study lacks novelty as many other previous studies reported bla-TEM and bla-CTXM in the poultry samples all over the globe.
