# Supplementary material for: Molecular detection and antimicrobial resistance profiles of Extended-Spectrum Beta-Lactamase (ESBL) producing Escherichia coli in broiler chicken farms in Malaysia
Source: PLoS One. 2023 May 19;18(5):e0285743. doi: 10.1371/journal.pone.0285743 (PMC10198488; doi:10.1371/journal.pone.0285743)
Supplement: Attachment — Submitted filename: Response to Reviewers_2.docx [file pone.0285743.s005.docx]

**Reviewer #2:**

**Comment-1**

**1**.Page 11, Line 102-03: Please provide brief description how Multiple antibiotic resistance (MAR) index was analyzed.

Response-1

Thank you for the comment and suggestion, MAR index calculation described in page 5, line 102-03

Multiple antibiotic resistance (MAR) index was calculated by dividing the number of resistant antibiotics which are resistance to an organism to the total number of antibiotics tested

$$MAR index= \frac{Number of antimicrobials to which the isolate showed resistance}{Number of total antibiotics exposed to the isolate}$$

**Comment-2**
**2.** Some anomalies of reference citation were noticed. Reference numbers # 27 and #28 are the publications shown differently. Similar anomalies might be present. Please provide some other stronger references that attest 'Pho-gene" specific E. coli detection. Please include 16s rDNA gene sequence to confirm E. coli.

Response-2

Thank you for the correction, the anomalies of references 27 and 28 are corrected in the manuscript. In addition to Pho gene, we used E.coli primer to confirm E.coli detection. We checked all references and added most relevant reference into the manuscript.

**Comment-3**

**3.** Please provide a supplementary figure stating cultural, biochemical characteristics, antimicrobial susceptibility analyses, and Polymerase chain reaction results for different genes described in the manuscript.

Response-3

Thank you for the comment. We have included Ten laboratory supplementary figures in a separate file as per requested.

**Comment-4**

4. At page 14, line 140-41, described higher resistance of the E. coli isolates against meropenem (28.9%) than that of imipenem (17.5%) is unusual. Need to reinvestigate the findings.
Response-4

Yes, we agree that many literatures on animal originated *E. coli* mostly reported higher resistance to imipenem than to meropenem; however, the isolated *E. coli* in our study showed more resistance to meropenem than imipenem. As part of the overall research, we have done further confirmation of the resulst using modified Carbapenem inactivation method for the phenotypic detection of carbapenem resistance, however, our primary focus in this specific manuscript was on ESBL producing E. coli, hence we did not put much detail on other resistance aspects of the E. coli isolates except for screening using the antimicrobial susceptibility profile of the isolated E. coli.

**Comment-5**

**5.** Table 2 has described both the resistant and susceptible frequency. As resistant frequency will be the complementary of the susceptible frequency considering the total number tested, describing both frequencies is unnecessary.

Response-5: Thank you for the suggestion correction have been made accordingly.

**Comment-6**
6. Page 15-16, line 150-162, as part of ESBL gene analysis only two genes, bla-TEM and bla-CTXM were analyzed.

a) Some more genes should be included to draw conclusions about ESBL associated resistance.

Response-6a.thank you for the comment, yes, we agree that there are additional ESBL genes such as *bla_SHV_*; however, the most reported ESBL genes from animal originated *E.coli* specifically in poultry are bla*_CTX-M_* and *bla-_TEM_* _.A_

**Here are some references.**

1. Gundran RS, Cardenio PA, Villanueva MA, Sison FB, Benigno CC, Kreausukon K, et al. Prevalence and distribution of blaCTX-M, blaSHV, blaTEM genes in extended- spectrum beta- lactamase- producing E. coli isolates from broiler farms in the Philippines. BMC Vet Res. 2019;15(1):227. Epub 2019/07/07. doi: 10.1186/s12917-019-1975-9. PubMed PMID: 31277658; PubMed Central PMCID: PMCPMC6612079.

2. Hiroi M, Yamazaki F, Harada T, Takahashi N, Iida N, Noda Y, et al. Prevalence of extended-spectrum beta-lactamase-producing Escherichia coli and Klebsiella pneumoniae in food-producing animals. J Vet Med Sci. 2012;74(2):189-95. Epub 2011/10/08. doi: 10.1292/jvms.11-0372. PubMed PMID: 21979457.

3. Wu C, Wang Y, Shi X, Wang S, Ren H, Shen Z, et al. Rapid rise of the ESBL and mcr-1 genes in Escherichia coli of chicken origin in China, 2008-2014. Emerg Microbes Infect. 2018;7(1):30. Epub 2018/03/15. doi: 10.1038/s41426-018-0033-1. PubMed PMID: 29535301; PubMed Central PMCID: PMCPMC5849743.

b) There was no description about the type of bla-CTXM (type-9/ 15?).

Response-6b thank you for the suggestion, we focus on the analyzsis of the bla-CTX-M group only not specific group.

c) There should be statistical analyses to see the association of the bla-TEM and bla-CTXM genes with the bacterial phenotypic resistance phenomena.

Response-6c Correlation have been made to see association of the bla-TEM and bla-CTXM genes with the bacterial phenotypic resistance and the heatmap plot is added to the manuscript
d) Not clear why colistin resistance gene, mcr-1 was analyzed under the titleship of ESBL. Need justification at the introduction and discussion.

Response 6 d: thank you for the comment, to show the isolated *E. coli* were co-resistant to both critically important antimicrobial (ESBL and colistin). We are working on a project on Colistin resistance and ESBL producing *E. coli* in broiler chickens, this result is part of the project,
**Comment-7**

**7.** For performing AST, authors should use at least one or two ATCC controls to validate and interpret their result.

Response-7 Thank you for the suggestion, we used *E. coli* ATCC 25922 as a control strain, as stated in page 5, line100, *E. coli* ATCC 25922 was used as a control strain.
**Comment-8**

8. This study lacks novelty as many other previous studies reported bla-TEM and bla-CTXM in poultry samples all over the globe.

Response-8 Thank you for the comment yes it has been reported throughout the globe, but this is the first report from the study area on poultry samples, and we report genotypic and phenotypic resistance profile of the isolates with their corresponding sequence typing (MLST).
